# Maternal care experience and postpartum depressive symptoms among migrant and native in Portugal

**DOI:** 10.1093/eurpub/ckac130.087

**Published:** 2022-10-25

**Authors:** C Teixeira, S Santos, J Guerra, H Barros

**Affiliations:** 1 EPIUnit, Instituto de Saúde Pública, Universidade do Porto, Porto, Portugal; 2 Escola Superior de Saúde, Instituto Politécnico de Bragança, Bragança, Portugal; 3 Departamento de Psiquiatria, Centro Hospitalar Universitário do Porto, Porto, Portugal; 4 Ciências de Saúde Publica e Forenses, Faculdade de Medicina, Universidade do Porto, Porto, Portugal

## Abstract

**Background:**

Migration is a risk factor for both, poor maternal experience with healthcare services (MEHCS) and postpartum depressive symptoms (PPDS), a matter of concern due to their adverse consequences. We aimed to assess the association between MEHCS and PPDS taking into account the migration status.

**Methods:**

This is part of a population-based study (baMBINO project), enrolling native (PT; n = 1568), permanent migrant (PM; n = 676) and temporary migrant (TM; n = 757) women recruited at delivery (2017-2019) in 32 Portuguese public hospitals. MEHCS was assessed based on 39 items of the Migrant Friendly Maternal Care Questionnaire asking about how women have experienced maternal care during pregnancy, during delivery and after birth. Items were grouped into 9 components each one assessing a different issue of MEHCS. For each component women were classified as having “good” or “less than good” experience. PPDS were assessed using the Edinburgh Postnatal Depression Scale (cut-off≥12). Multivariate logistic regression model was fitted to estimate the association between MEHCS and PPDS. Adjusted odds ratio (aOR) and respective 95% confidence interval were obtained.

**Results:**

PPDS were reported by 3.8%, 5.8% and 8.2% of PT, PM and TM women, respectively (p < 0.001). After adjustment, 4 out 9 components of MEHCS appeared related with PPDS, such that women reporting less than good experience with “understanding information” (aOR=1.72 95%CI:1.14-2.60), “decisions according to maternal wishes” (aOR=1.56 95% CI:1.04-2.34), “time waiting for healthcare” (aOR=1.50 95%CI:1.04-2.18) and “healthcare provider's attitudes during pregnancy” (aOR=1.58 95%CI:1.01-2.47) showed higher odds of PPDS than women reporting good experience.

**Conclusions:**

Further than the migration status, poor experience with some issues of maternal care seems play a role in the risk of PPDS

**Key messages:**

- Awareness should be given to maternal care experience.
- Public health strategies should address factors underlying a positive maternal experience.

